# Interactive Effects of Indigestible Carbohydrates, Protein Type, and Protein Level on Biomarkers of Large Intestine Health in Rats

**DOI:** 10.1371/journal.pone.0142176

**Published:** 2015-11-04

**Authors:** Marcin Taciak, Marcin Barszcz, Anna Tuśnio, Barbara Pastuszewska

**Affiliations:** Department of Monogastric Nutrition, The Kielanowski Institute of Animal Physiology and Nutrition Polish Academy of Sciences, Jabłonna, Poland; Western University of Health Sciences, UNITED STATES

## Abstract

The effects of indigestible carbohydrates, protein type, and protein level on large intestine health were examined in rats. For 21 days, 12 groups of six 12-week-old male Wistar rats were fed diets with casein (CAS), or potato protein concentrate (PPC), providing 14% (lower protein level; LP), or 20% (higher protein level; HP) protein, and containing cellulose, resistant potato starch, or pectin. Fermentation end-products, pH, and β-glucuronidase levels in cecal digesta, and ammonia levels in colonic digesta were determined. Cecal digesta, tissue weights, cecal and colon morphology, and colonocyte DNA damage were also analyzed. Digesta pH was lower, whereas relative mass of cecal tissue and digesta were higher in rats fed pectin diets than in those fed cellulose. Cecal parameters were greater in rats fed PPC and HP diets than in those fed CAS and LP diets, respectively. Short-chain fatty acid (SCFA) concentrations were unaffected by protein or carbohydrate type. Total SCFA, acetic acid, and propionic acid concentrations were greater in rats fed LP diets than in those fed HP. Cecal pool of isobutyric and isovaleric acids was greater in rats fed PPC than in those fed CAS diets. PPC diets decreased phenol concentration and increased ammonia concentration in cecal and colonic digesta, respectively. Cecal crypt depth was greater in rats fed PPC and HP diets, and was unaffected by carbohydrates; whereas colonic crypt depth was greater in rats fed cellulose. Myenteron thickness in the cecum was unaffected by nutrition, but was greater in the colon of rats fed cellulose. Colonocyte DNA damage was greater in rats fed LP diets than in those fed HP diets, and was unaffected by carbohydrate or protein type. It was found that nutritional factors decreasing cecal digesta weight contribute to greater phenol production, increased DNA damage, and reduced ammonia concentration in the colon.

## Introduction

Diet is a major environmental factor affecting the health status of the large intestine and is strongly involved in the pathogenesis of colorectal cancer, which is one of the most frequent cancers in western populations of human [1]. Colonic epithelium is exposed to many toxic agents of endogenous and exogenous origin that can induce DNA damage, which is found to be the first step in carcinogenesis [2, 3]. Endogenous factors include by-products of cellular metabolism and carcinogenic substances circulating in the blood, whereas intestinal digesta may be considered a source of exogenous carcinogens. The amounts and types of potential carcinogens in the digesta are dependent on dietary habits. Epidemiological data show that diets rich in protein increase the risk of colorectal cancer, whereas diets comprising fruits, vegetables, and cereals reduce the incidence of diseases of the large intestine [4]. Adverse effects of high protein intake are associated with bacterial metabolism of protein in the colon. Approximately 12 g of protein enters the human large intestine daily, at least half of which is of dietary origin. Other sources of protein include enzymes, mucus, and exfoliated epithelial cells. In the large intestine, protein is fermented by bacteria, which produce short-chain fatty acids (SCFA), branched-chain fatty acids (BCFA), and potentially toxic compounds such as ammonia, amines, phenols, N-nitroso compounds, and sulfur metabolites [5]. The extent of the bacterial fermentation of protein depends not only on the amount of protein reaching the colon, but also on the carbohydrate-nitrogen ratio. When this ratio decreases, due to depletion of fermentable carbohydrates, intestinal microbiota catabolize protein [6]. Proteolysis in the large intestine may be affected by the presence of indigestible carbohydrates, which are components of dietary fiber. Carbohydrates differ in their chemical properties and thus in the pattern and extent of fermentation. Pectin is a highly soluble dietary fiber that is rapidly fermented in the large intestine, resulting in a high concentration of acetic acid. Cellulose, on the other hand, is an insoluble component of dietary fiber that is very resistant to fermentation. Resistant starch is a good source of butyric acid, but its fermentation seems to depend on its source and structure. The substrate types available to microbiota may affect the carbohydrate-nitrogen ratio and the site of fermentation, which is important for large intestinal health, since most cancers develops in the distal colon [7]. The negative effects of high protein diets on colonocyte DNA in rats may be reduced by the inclusion of resistant starch in the diet [4, 8]. Other indigestible carbohydrates may however, show other effects depending on the source (animal or plant origin), digestibility, and level of protein in the diet. Interactions between indigestible carbohydrates and protein types and levels may modify the fermentation profile in the large intestine and thereby affect its health. Therefore, the aim of the present study was to determine the effects of protein type (casein or potato protein) and level (14% or 20%) on the cecal fermentation profile, large intestinal morphology, and colonocyte DNA damage in rats fed diets with various indigestible carbohydrates (cellulose, pectin, and resistant potato starch). Potato protein was selected for its lower ileal digestibility, in comparison to casein, which makes more nitrogen available for hindgut microflora.

## Materials and Methods

### Animals and diets

The study design, animal care, and experimental procedures were approved by the Third Local Animal Experimentation Ethics Committee (resolution number 34/2007, Warsaw University of Life Sciences-SGGW, Warsaw, Poland) in accordance with the principles of laboratory animal care of the European Union and Polish Law on Animal Protection. The study was conducted on 72 12-week-old male Crl:W (Han) Wistar rats of initial body weight 330 g, divided into 12 groups (n = 6). Animals were fed diets containing casein (CAS) or potato protein concentrate (PPC), providing either 14% (lower protein level, LP) or 20% (higher protein level, HP) crude protein, and supplemented with 10% indigestible carbohydrate i.e. cellulose, resistant potato starch (RPS), or pectin. Composition of the experimental diets is shown in Table 1. Rats were kept individually in wire-bottom metabolic cages, under controlled conditions of 22 ± 1°C and 12/12 h dark/light cycles, with free access to feed and water. Body weight and feed intake measurements were recorded weekly. After 21 days, rats were sacrificed by CO_2_ asphyxiation. Ceca were resected, emptied, and weighed. Cecal digesta was taken for pH measurements and analyses of SCFA, phenol, p-cresol, and bacterial β-glucuronidase. Cecal digesta samples were stored at -20°C for analyses of organic acids and phenolic compounds, and at -80°C for the β-glucuronidase assay. Tissue samples from the cecum and colon were taken, rinsed with 0.9% NaCl, and placed in Bouin’s fixative for histological examinations. Approximately 10 cm of the distal colon was resected for isolation of colonocytes and the comet assay, whereas colonic contents were sampled and stored at -20°C for ammonia determination.

**Table 1:** Composition of Experimental Diets (g/kg).

	Casein						Potato protein					
	14% of protein			20% of protein			14% of protein			20% of protein		
Ingredient	Cellulose	Potato starch	Pectin	Cellulose	Potato starch	Pectin	Cellulose	Potato starch	Pectin	Cellulose	Potato starch	Pectin
Casein	173	173	173	248	248	248	-	-	-	-	-	-
Potato protein concentrate	-	-	-	-	-	-	184	184	184	263	263	263
Cellulose	100	-	-	100	-	-	100	-	-	100	-	-
Resistant potato starch	-	100	-	-	100	-	-	100	-	-	100	-
Pectin	-	-	100	-	-	100	-	-	100	-	-	100
Corn starch	517	517	517	442	442	442	506	506	506	427	427	427
Sucrose	120	120	120	120	120	120	120	120	120	120	120	120
Soybean oil	43	43	43	43	43	43	43	43	43	43	43	43
Minerals^1^	35	35	35	35	35	35	35	35	35	35	35	35
Vitamins^2^	10	10	10	10	10	10	10	10	10	10	10	10
Choline chloride	2	2	2	2	2	2	2	2	2	2	2	2

^1^AIN-93G Mineral Mix, MP Biomedical, Inc., Eschwege, Germany. Mixture contains the following components: (%)—calcium carbonate 35.7; monopotassium phosphate 19.6; potassium citrate monohydrate 7.078; sodium chloride 7.4; potassium sulfate 4.66; magnesium oxide 2.4; ferric citrate 0.606; zinc carbonate 0.165; manganese carbonate 0.063; copper carbonate 0.03; potassium iodate 0.001; sodium selenate, anhydrous 0.00103; ammonium molybdate^.^4H_2_O 0.000795; sodium metasilicate^.^9H_2_O 0.145; chromium potassium sulfate^.^12H_2_O 0.0275; lithium chloride 0.00174; boric acid 0.008145; sodium fluoride 0.00635; nickel carbonate 0.00318; ammonium vanadate 0.00066; powdered sugar 22.1.

^2^AIN-93-VX Vitamin Mix, MP Biomedicals, Inc., Eschwege, Germany. Mixture contains the following components: (g/kg)–nicotinic acid 3.00; D-calcium pantothenate1.60; pyridoxine HCl 0.70; thiamine HCl 0.60; riboflavin 0.60; folic acid 0.20; D-biotin 0.02; vitamin B_12_ (0.1% triturated in mannitol) 2.50; α-tocopherol powder (250 U/gm) 30.00; vitamin A palmitate (250,000 U/gm) 1.60; vitamin D_3_ (400,000 U/gm) 0.25; phylloquinone 0.075; powdered sucrose 959.655.

### Measurement of digesta pH and SCFA

Digesta pH was measured by a WTW pH/340 pH-meter (WTW GmbH, Weilheim, Germany) and SCFA analysis was performed using the HP 5890 Series II gas chromatograph (Hewlett-Packard, Waldbronn, Germany) with isocaproic acid as the internal standard [9].

### Analysis of phenol and p-cresol

Phenol and p-cresol concentrations in cecal digesta were assessed based on previously described methods [10] with the following modifications. Each sample (1.0 g) was mixed with 1.5 mL methanol and incubated for 1 h on ice with frequent vortexing. After incubation, samples were centrifuged at 12 000 rpm for 15 min at 4°C. The supernatant (500 μL) was transferred to vials and mixed with 15 μL 5-methylindole as the internal standard. It was further analyzed using the HP 5890 Series II gas chromatograph (Hewlett-Packard, Waldbronn, Germany) with a flame-ionization detector and Supelco Nukol™ (Supelco, Bellefonte, USA) fused silica capillary column (60 m × 0.32 mm I.D.; 0.25 μm). The oven temperature was set at 55°C for 1 min, then raised to 180°C at a rate of 20°C/min, and held for 25 min. The temperature was then raised to 200°C at 20°C/min, and held for 27 min. Injector and detector temperatures were 220°C and helium was used as the carrier gas. The total run time was 60.25 min. Phenol and p-cresol concentrations were calculated based on standard curves.

### Analysis of β-glucuronidase

β-glucuronidase activity in cecal digesta samples was quantified spectrophotometrically [9], based on the amount of phenolphthalein released from the substrate (phenolphthalein *β*-D-glucuronide). The absorbance was measured at 540 nm using a Unicam UV 300 spectrophotometer (Thermo-Spectronic, Cambridge, UK).

### Analysis of ammonia

Concentration of ammonia in colonic contents was quantified spectrophotometrically based on the reaction of the ammonium ion with Nessler’s reagent. The sample (0.5 g) was mixed with 2 mL of ultra pure water, acidified with 1M HCl to adjust the pH to 5.0–6.0, and homogenized for 30 s at high speed. Samples were then centrifuged at 10 000 rpm for 10 min at 4°C. The supernatant (1.0 mL) was transferred into a new tube and incubated with approximately 0.18 g of basic magnesium carbonate for 20 min at room temperature, with frequent mixing to remove ferrous ions, which could distort the results of the analysis. After incubation, samples were centrifuged at 3000 rpm for 10 min. The following steps were then performed using Maxmat PL multidisciplinary diagnostic platform (Erba Diagnostics France SARL, Montpellier, France). The supernatant (5 μL) was placed on a micro titer plate, diluted with 220 μL ultra pure water and mixed with 25 μL Nessler’s reagent. The absorbance was measured immediately at 425 nm and ammonium concentration was calculated from the standard curve prepared using ammonium chloride solution.

### Cecum and colon histology

Tissue samples were embedded in paraffin, sliced into 5 μm sections, and stained with hematoxylin and eosin. Crypt depth and myenteron thickness (15 measurements per slide, two slides per sample) were measured using the Zeiss Axio Star Plus light microscope (Carl Zeiss, Göttingen, Germany) and the Axio Vision LE Release 4.5 image analysis software (Carl Zeiss, 2002–2005).

### Colonocyte isolation and alkaline comet assay

Colonocytes were isolated according to a modified method previously described [3]. After rinsing with 0.9% NaCl, distal colon samples were successively washed and kept on ice in solution A (96 mM NaCl, 27 mM Na-citrate, 1.5 mM KCl, 0.8 mM KH_2_PO_4_, 5.6 mM Na_2_HPO_4_, 25 mM NaHCO_3_, 1 mM dithiothreitol, and 2.5 mM L-glutamine, gassed with carbogen) until all samples were collected, but no longer than one hour. The following steps were then performed under dimmed light to prevent UV-induced DNA damage. Colon samples were closed with a clip at one end, filled with approximately 5 mL of solution A, closed with another clip from the other side, and incubated in a shaking water bath for 10 min at 30°C in solution A. After incubation, solution A was discarded and replaced by 5 mL of solution B (1 mM EDTA, 115 mM NaCl, 25 mM NaHCO_3_, 2.4 mM K_2_HPO_4_, 0.4 mM KH_2_PO_4_, 1 mM dithiothreitol, and 2.5 mM L-glutamine, gassed with carbogen) and incubated for 15 min at 30°C in solution B in a shaking water bath. After incubation, samples were placed on Petri dishes and gently palpated for 3 min. Colonic contents were collected and centrifuged for 7 min at 1400 rpm, and the pellets thereby obtained were washed by ice cold phenol red-free Dulbecco’s Modified Eagle Medium (DMEM) containing 1% L-glutamine and 4 mM CaCl_2_, and centrifuged again. The pellets were resuspended in Hank’s Balanced Salt Solution (HBSS) with 0.1 mg/mL collagenase type II and incubated for 30 min at 30°C. Samples were then centrifuged again to remove collagenase and resuspended in DMEM. Colonic epithelial cells were resuspended in 5 mL of ice cold 1X PBS, pH 7.4. Cell viability was checked by trypan blue exclusion using a hemocytometer, and efficiency of cell isolation was determined by histological examination.

The alkaline comet assay was performed according to manufacturer instructions using Trevigen CometSlide^TM^ (Trevigen, Gaithersburg, MD, USA) and horizontal electrophoresis unit. Comet images were visualized by ethidium bromide (2 μg/mL) staining and analyzed at 40X magnification, using an Olympus BX51 fluorescent microscope (Olympus Corp., Tokyo, Japan) equipped with a 510–550 nm excitation filter and a 590 nm barrier filter. Comets were analyzed using Cell^D^ Imaging Software (Olympus Soft Imaging Solutions GmbH, Munster, Germany). For each sample, 75 randomly selected comets were assigned to five classes from 0 (undamaged) to 4 (maximally damaged). “Tail intensity” was expressed in arbitrary units [11] and ranged from 0 to 400.

### Statistical analysis

Data were analyzed by three-way analysis of variance (ANOVA), followed by *post hoc* Tukey’s HSD test for determination of differences between treatments. In addition, Pearson’s correlation coefficients between analyzed parameters were calculated. All analyses were performed using the STATGRAPHICS^®^ Centurion XVI ver. 16.1.03 statistical package (StatPoint Technologies, Inc., Warrenton, Virginia, USA). The effects and correlations were considered significant at *P* ≤ 0.05.

## Results

### Body weight gain and cecal parameters

Experimental diets affected parameters related to cecal fermentation, including relative weight of cecal digesta and tissue, and pH of cecal digesta (Table 2), but did not affect feed intake (data not shown) or body weight gain of rats. Relative weight of cecal digesta was greater in rats fed PPC and HP, than in those fed CAS and LP diets, respectively (*P* = 0.001). Relative weight of cecal tissue was also greater in rats fed PPC than CAS diet (*P* = 0.002), but was not influenced by protein level. The type of indigestible carbohydrates affected both cecal digesta and tissue weights, which were greater in rats fed pectin than in those fed cellulose and RPS (*P* ≤ 0.001). However, the effect of carbohydrates on cecal tissue weight was found only in rats fed CAS, and not in those fed PPC (*P* = 0.017). Rats fed pectin diets had similar cecal tissue weights regardless of protein type; whereas rats fed RPS and cellulose with PPC had greater cecal tissue weights than animals fed CAS diets.

**Table 2:** Body Weight Gain and Cecal Variables in Rats Fed the Experimental Diets. Data are presented as mean values ± SEM.

Protein level	Indigestible carbohydrates	Body weight gain (g)	Cecal tissue (% of body weight)	Cecal digesta (% of body weight)	pH
		**Casein**			
14%	Cellulose	22.9 ± 5.3	0.23 ± 0.03	0.69 ± 0.08	7.56 ± 0.05
	Potato starch	19.6 ± 3.1	0.31 ± 0.04	0.83 ± 0.12	6.33 ± 0.17
	Pectin	26.9 ± 2.9	0.34 ± 0.02	0.94 ± 0.07	6.87 ± 0.10
20%	Cellulose	20.5 ± 3.3	0.22 ± 0.01	0.71 ± 0.05	7.40 ± 0.04
	Potato starch	30.4 ± 4.1	0.25 ± 0.01	0.96 ± 0.08	6.53 ± 0.04
	Pectin	23.4 ± 3.5	0.34 ± 0.02	1.30 ± 0.11	6.31 ± 0.07
		**Potato protein**			
14%	Cellulose	23.8 ± 2.8	0.31 ± 0.02	0.86 ± 0.10	7.05 ± 0.10
	Potato starch	22.0 ± 3.4	0.31 ± 0.02	0.87 ± 0.09	6.53 ± 0.25
	Pectin	20.9 ± 5.0	0.34 ± 0.02	1.23 ± 0.09	6.42 ± 0.11
20%	Cellulose	21.9 ± 6.1	0.31 ± 0.01	1.26 ± 0.09	6.98 ± 0.10
	Potato starch	27.0 ± 4.5	0.34 ± 0.02	1.25 ± 0.14	6.86 ± 0.23
	Pectin	19.1 ± 4.7	0.32 ± 0.02	1.12 ± 0.10	6.65 ± 0.09
Main effects (*P* values)					
Protein type		0.5415	0.002	0.001	0.253
Protein level		0.6799	0.595	0.001	0.956
Carbohydrates		0.6807	< 0.001	0.001	< 0.001
Protein type x Protein level		0.8031	0.256	0.631	0.026
Protein type x Carbohydrates		0.5482	0.017	0.089	0.001
Protein level x Carbohydrates		0.1631	0.910	0.618	0.043
All		0.8087	0.165	0.005	0.112

The pH of cecal digesta was higher in rats fed cellulose than in those fed pectin and RPS, and was affected by interactions between experimental factors. Rats fed PPC or HP diets had lower digesta pH than rats on CAS or LP diets except rats fed PPC or HP diets with RPS (*P* = 0.001 and *P* = 0.043). There was also significant interaction between protein type and level (*P* = 0.026). Diets with a lower protein level provided by casein increased pH, whereas the same amount of potato protein lowered pH in comparison to a higher protein level. Relative cecal weight was positively correlated with cecal content weight (*r* = 0.74, *P* = 0.005) and inversely correlated with digesta pH (*r* = –0.63, *P* = 0.027).

### Short-chain fatty acids

Concentrations of acetic and propionic acids, as well as the sum of SCFAs were greater in the cecal digesta of rats fed LP than HP diets (*P* = 0.043, *P* = 0.004, and *P* = 0.022, respectively) (Table 3). Indigestible carbohydrates and protein type had no effect on SCFA concentration, but significant interaction between protein type, protein level, and carbohydrates affected concentrations of acetic (*P* = 0.022), isobutyric (*P* = 0.004), isovaleric (*P* = 0.001), and valeric acids (*P* = 0.047), as well as the sum of SCFAs (*P* = 0.039). Relative weight of cecal digesta was inversely correlated with propionic acid (*r* = –0.68, *P* = 0.014), and butyric acid concentration (*r* = –0.75, *P* = 0.005), and tended to be inversely correlated with the total SCFA concentration (*r* = –0.55, *P* = 0.060). Cecal pool of butyric acid was directly proportional to digesta pH (*r* = 0.60, *P* = 0.038).

**Table 3:** Concentrations (μmol/g) of Short-Chain Fatty Acids in Cecal Digesta of Rats. Data are presented as mean values ± SEM.

Protein level	Indigestible carbohydrates	Acetic	Propionic	Isobutyric	Butyric	Isovaleric	Valeric	Total SCFA
		**Casein**						
14%	Cellulose	45.2 ± 11.0	21.3 ± 4.7	1.08 ± 0.16	6.8 ± 2.0	0.70 ± 0.16	0.82 ± 0.19	75.9 ± 16.0
	Potato starch	35.5 ± 3.2	16.4 ± 2.3	1.22 ± 0.19	7.7 ± 1.6	0.82 ± 0.15	0.83 ± 0.15	62.5 ± 5.7
	Pectin	39.7 ± 6.1	16.9 ± 2.4	1.18 ± 0.19	6.9 ± 1.0	0.91 ± 0.23	0.78 ± 0.16	66.4 ± 9.5
20%	Cellulose	33.7 ± 5.8	15.3 ± 2.4	0.97 ± 0.09	6.4 ± 1.8	0.56 ± 0.11	0.72 ± 0.16	57.6 ± 8.6
	Potato starch	36.6 ± 8.7	13.1 ± 2.8	1.24 ± 0.20	6.9 ± 3.0	0.74 ± 0.16	0.64 ± 0.13	59.2 ± 14.4
	Pectin	25.3 ± 7.5	8.0 ± 2.1	0.66 ± 0.08	3.2 ± 0.9	0.43 ± 0.09	0.34 ± 0.10	38.0 ± 9.9
		**Potato protein**						
14%	Cellulose	47.2 ± 7.0	16.9 ± 2.5	1.13 ± 0.17	7.0 ± 1.0	0.79 ± 0.22	0.68 ± 0.14	73.8 ± 9.8
	Potato starch	69.9 ± 17.8	25.2 ± 7.9	1.71 ± 0.45	7.3 ± 2.1	1.32 ± 0.38	1.23 ± 0.37	106.6 ± 28.7
	Pectin	27.1 ± 4.2	13.4 ± 2.2	0.67 ± 0.05	4.3 ± 1.2	0.30 ± 0.08	0.36 ± 0.09	46.2 ± 6.7
20%	Cellulose	43.0 ± 5.7	14.3 ± 1.1	1.15 ± 0.08	6.2 ± 1.0	0.97 ± 0.14	0.99 ± 0.30	66.6 ± 7.0
	Potato starch	32.1 ± 3.4	12.9 ± 1.5	0.94 ± 0.23	5.6 ± 1.6	0.52 ± 0.16	0.52 ± 0.10	52.6 ± 5.8
	Pectin	36.5 ± 3.8	12.4 ± 0.8	1.27 ± 0.09	4.5 ± 0.4	1.14 ± 0.17	0.74 ± 0.19	56.5 ± 5.0
Main effects (*P* values)								
Protein type		0.156	0.719	0.464	0.582	0.180	0.559	0.322
Protein level		0.043	0.004	0.263	0.200	0.451	0.260	0.022
Carbohydrates		0.098	0.119	0.060	0.135	0.496	0.111	0.074
Protein type x Protein level		0.780	0.840	0.507	0.659	0.168	0.279	0.983
Protein type x Carbohydrates		0.387	0.333	0.970	0.916	0.755	0.852	0.503
Protein level x Carbohydrates		0.370	0.726	0.281	0.887	0.061	0.113	0.494
All		0.022	0.185	0.004	0.523	0.001	0.047	0.039

Feeding PPC diets increased the cecal pool of acetic (*P* = 0.025), isobutyric (*P* = 0.010), and isovaleric acids (*P* = 0.022) compared to feeding CAS diets, whereas rats fed HP diets had a greater pool of isobutyric (*P* = 0.009) and isovaleric acids (*P* = 0.052) than rats fed LP diets (Table 4). Interaction between protein type and protein level significantly affected cecal pools of isoacids (*P* = 0.005) and valeric acid (*P* = 0.014), which were similar in rats on CAS diets regardless of protein level, but on PPC diets the pools were greater at higher than lower protein level. Cecal pools of isoacids were also affected by the interaction between protein type and carbohydrates. The pool of isobutyric acid in rats fed CAS diets was greatest when RPS was used as an indigestible carbohydrate, and least when pectin was used; whereas the isobutyric acid pool in rats fed PPC diets was greatest in those fed cellulose, and least in those fed RPS (*P* = 0.008). Rats fed CAS diets with cellulose had a smaller isovaleric acid pool compared to those fed RPS and pectin. In contrast, rats fed PPC diets with cellulose, had a larger isovaleric acid pool compared to those fed other carbohydrates (*P* = 0.033). There was also a significant effect of interaction between all experimental factors on cecal pools of isobutyric and isovaleric acids (*P* = 0.010 and *P* = 0.003, respectively).

**Table 4:** Cecal Pool (μmol/cecum) of Short-Chain Fatty Acids in Rats. Data are presented as mean values ± SEM.

Protein level	Indigestible carbohydrates	Acetic	Propionic	Isobutyric	Butyric	Isovaleric	Valeric	Total SCFA
		**Casein**						
14%	Cellulose	97.3 ± 15.4	46.1 ± 7.1	2.5 ± 0.3	16.4 ± 6.1	1.6 ± 0.3	1.8 ± 0.3	165.8 ± 22.4
	Potato starch	108.8 ± 21.0	52.6 ± 15.4	3.3 ± 0.2	25.4 ± 8.8	2.2 ± 0.2	2.5 ± 0.7	194.7 ± 43.8
	Pectin	129.2 ± 14.0	55.4 ± 6.1	3.9 ± 0.6	22.7 ± 2.9	3.0 ± 0.7	2.6 ± 0.5	216.8 ± 22.5
20%	Cellulose	83.1 ± 13.0	38.4 ± 6.4	2.4 ± 0.1	16.8 ± 6.1	1.3 ± 0.2	1.7 ± 0.3	143.7 ± 21.5
	Potato starch	124.4 ± 30.9	44.6 ± 10.2	4.1 ± 0.5	24.4 ± 11.0	2.5 ± 0.4	2.2 ± 0.4	202.3 ± 52.3
	Pectin	124.3 ± 43.5	39.7 ± 12.5	3.0 ± 0.4	16.5 ± 5.3	2.1 ± 0.5	1.6 ± 0.5	187.2 ± 58.5
		**Potato protein**						
14%	Cellulose	133.2 ± 17.2	48.7 ± 6.4	3.2 ± 0.6	20.8 ± 3.2	2.3 ± 0.7	2.0 ± 0.5	210.3 ± 24.7
	Potato starch	144.8 ± 21.0	49.4 ± 8.2	3.5 ± 0.4	14.4 ± 1.9	2.7 ± 0.6	2.5 ± 0.5	217.3 ± 30.8
	Pectin	109.3 ± 13.4	55.1 ± 9.4	2.8 ± 0.2	17.8 ± 5.4	1.2 ± 0.3	1.5 ± 0.4	187.7 ± 24.5
20%	Cellulose	189.1 ± 31.4	62.4 ± 6.5	4.9 ± 0.4	28.5 ± 6.5	4.3 ± 0.7	4.1 ± 1.0	293.4 ± 42.0
	Potato starch	133.9 ± 15.1	53.2 ± 4.5	3.7 ± 0.5	24.1 ± 7.3	2.0 ± 0.4	2.2 ± 0.3	219.1 ± 22.9
	Pectin	139.4 ± 16.1	47.8 ± 5.2	4.8 ± 0.4	17.1 ± 2.0	4.3 ± 0.7	2.9 ± 0.7	216.4 ± 23.2
Main effects (*P* values)								
Protein type		0.025	0.191	0.010	0.980	0.022	0.147	0.057
Protein level		0.370	0.485	0.009	0.643	0.052	0.298	0.566
Carbohydrates		0.986	0.985	0.319	0.715	0.640	0.793	0.964
Protein type x Protein level		0.325	0.174	0.005	0.273	0.005	0.014	0.196
Protein type x Carbohydrates		0.079	0.645	0.008	0.270	0.033	0.160	0.123
Protein level x Carbohydrates		0.849	0.497	0.884	0.598	0.165	0.220	0.797
All		0.326	0.859	0.010	0.956	0.003	0.210	0.530

### Phenol and p-cresol

Protein type affected phenol concentration (Table 5), which was greater in the cecum of rats fed CAS than in those fed PPC diets (*P* = 0.029). Experimental factors had no effect on phenol or p-cresol concentrations. The concentration of p-cresol was positively correlated with cecal pool of valeric (*r* = 0.67, *P* = 0.018) and isovaleric acids (*r* = 0.67, *P* = 0.017). Cecal pool of p-cresol was significantly greater in rats fed PPC diets than in those fed CAS diets (*P* = 0.011).

**Table 5:** Concentrations and Cecal Pool of Phenolic Compounds and Activity of Bacterial β-glucuronidase in Cecal Digesta of Rats. Data are presented as mean values ± SEM

Protein level	Indigestible carbohydrates	Phenol, μmol/g	Phenol, μmol/cecum	p-cresol, μmol/g	p-cresol, μmol/cecum	β-glucuronidase, U/g	β-glucuronidase, U/cecum
		**Casein **					
14%	Cellulose	0.24 ± 0.08	0.54 ± 0.17	0.57 ± 0.14	1.54 ± 0.45	79.1 ± 11.6	200.7 ± 40.8
	Potato starch	0.33 ± 0.18	1.01 ± 0.45	0.69 ± 0.37	1.43 ± 0.46	71.9 ± 10.9	209.6 ± 43.1
	Pectin	0.14 ± 0.03	0.44 ± 0.08	0.51 ± 0.13	1.65 ± 0.32	123.6 ± 16.9	409.9 ± 45.5
20%	Cellulose	0.21 ± 0.06	0.47 ± 0.11	0.57 ± 0.12	1.34 ± 0.21	113.4 ± 27.2	279.0 ± 67.0
	Potato starch	0.13 ± 0.03	0.44 ± 0.07	0.46 ± 0.12	1.42 ± 0.27	47.0 ± 7.4	166.6 ± 33.2
	Pectin	0.11 ± 0.03	0.40 ± 0.13	0.37 ± 0.07	1.60 ± 0.21	109.0 ± 41.0	517.9 ± 193.2
		**Potato protein**					
14%	Cellulose	0.10 ± 0.02	0.34 ± 0.07	0.52 ± 0.15	1.78 ± 0.46	160.6 ± 66.8	554.2 ± 259.9
	Potato starch	0.16 ± 0.02	0.43 ± 0.08	0.65 ± 0.08	1.73 ± 0.33	33.8 ± 9.0	100.1 ± 34.3
	Pectin	0.10 ± 0.03	0.44 ± 0.11	0.46 ± 0.07	1.95 ± 0.34	51.7 ± 15.0	217.7 ± 73.0
20%	Cellulose	0.09 ± 0.01	0.37 ± 0.12	0.43 ± 0.09	1.84 ± 0.33	75.6 ± 17.2	331.8 ± 80.1
	Potato starch	0.11 ± 0.03	0.42 ± 0.11	0.78 ± 0.22	2.99 ± 0.66	85.2 ± 41.9	343.5 ± 144.9
	Pectin	0.12 ± 0.03	0.46 ± 0.12	0.76 ± 0.20	2.74 ± 0.68	174.2 ± 71.1	635.3 ± 247.7
Main effects (*P* values)							
Protein type		0.029	0.074	0.453	0.011	0.763	0.389
Protein level		0.156	0.298	0.962	0.230	0.495	0.210
Carbohydrates		0.311	0.378	0.488	0.481	0.064	0.044
Protein type x Protein level		0.297	0.232	0.213	0.125	0.444	0.523
Protein type x Carbohydrates		0.346	0.415	0.462	0.676	0.861	0.425
Protein level x Carbohydrates		0.277	0.280	0.808	0.549	0.288	0.212
All		0.717	0.365	0.451	0.729	0.033	0.192

### β-glucuronidase

β-glucuronidase activity, expressed per g of cecal digesta, was not affected by experimental factors (Table 5), however a significant effect of interaction between protein type, protein level, and carbohydrates was evident (*P* = 0.033). The lowest level of β-glucuronidase activity was in the cecum of rats fed diet with PPC with 14% crude protein supplemented with RPS, whereas the highest was in rats fed PPC with 20% crude protein supplemented with pectin. β-glucuronidase activity was directly proportional to the cecal pool of isobutyric (*r* = 0.58, *P* = 0.047) and isovaleric acids (*r* = 0.63, *P* = 0.028). Total β-glucuronidase activity, expressed per cecum, was significantly influenced by carbohydrates (*P* = 0.044), and was greater in rats fed pectin than in those fed RPS.

### Ammonia

**Fig 1:**
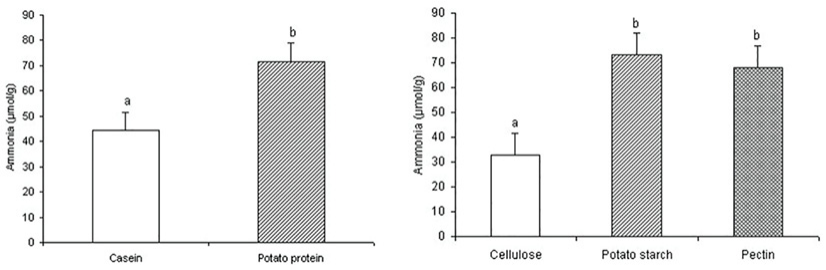
Ammonia concentration (μmol/g) in colonic digesta of rats. Error bars represent standard error of the mean. Means with a different letters differ significantly (P < 0.05).

Ammonia concentration in colonic digesta was greater in rats fed PPC compared to those fed CAS (*P* = 0.011), and in rats fed RPS and pectin compared to those fed cellulose (*P* = 0.004) (Fig 1). Protein level and interactions did not affect ammonia concentration. Ammonia concentration in colonic digesta was inversely correlated with cecal digesta pH (*r* = –0.71, *P* = 0.009), and positively correlated with the relative weight of cecal tissue (*r* = 0.72, *P* = 0.008).

### Crypt depth and myenteron thickness

Cecal crypt depth in rats fed PPC and LP diets was significantly increased compared to that of rats fed CAS (*P* = 0.029) and HP (*P* = 0.023) diets, respectively. Cecal crypt depth was not affected by carbohydrates and interactions (Table 6). In addition, experimental factors did not affect cecal myenteron thickness. Colonic crypt depth was affected only by the interaction between protein type and indigestible carbohydrates (*P* = 0.018). Rats fed PPC diets, with the exception of those fed PPC diets with cellulose, generally had shorter colon crypts than rats fed CAS diets. Colonic crypt depth was positively correlated with cecal butyric acid concentration (*r* = 0.61, *P* = 0.036). Colonic myenteron thickness was significantly greater in rats fed cellulose than in those fed pectin (*P* = 0.005). In addition, it was positively correlated with digesta pH (*r* = 0.82, *P* = 0.001); negatively correlated with the relative weight of cecal digesta (*r* = –0.66, *P* = 0.018) and total β-glucuronidase activity (*r* = –0.73, *P* = 0.007); and tended to be inversely proportional to colonic ammonia concentration (*r* = –0.55, *P* = 0.062). Interactions had no effect on colonic myenteron thickness.

**Table 6:** Morphological Parameters (μm) of Cecum and Colon and Colonocyte DNA Damage (tail intensity units) in Rats. Data are presented as mean values.

		Cecum		Colon		
Protein level	Indigestible carbohydrates	Crypt depth	Myenteron thickness	Crypt depth	Myenteron thickness	DNA damage
		**Casein**				
14%	Cellulose	239.6 ± 13.9	234.6 ± 35.3	249.5 ± 11.2	186.8 ± 8.1	307.8 ± 20.3
	Potato starch	250.1 ± 9.0	227.3 ± 19.9	275.3 ± 23.2	158.3 ± 13.1	298.0 ± 20.6
	Pectin	241.3 ± 9.8	223.1 ± 12.2	251.3 ± 6.5	156.0 ± 10.5	223.2 ± 24.1
20%	Cellulose	217.0 ± 8.5	203.6 ± 11.1	246.1 ± 6.4	185.9 ± 18.8	225.8 ± 28.2
	Potato starch	235.1 ± 12.5	207.8 ±11.7	247.9 ± 12.8	158.5 ± 8.6	222.5 ± 18.3
	Pectin	215.8 ± 7.5	220.5 ± 12.6	241.6 ± 10.3	124.8 ± 12.2	237.8 ± 32.4
		**Potato protein**				
14%	Cellulose	259.0 ± 10.2	253.6 ± 34.8	259.5 ± 9.4	163.0 ± 12.2	287.5 ± 29.4
	Potato starch	246.1 ± 7.8	212.8 ± 11.3	242.8 ± 14.0	147.4 ± 7.6	246.0 ± 14.5
	Pectin	248.0 ± 6.6	213.6 ± 9.1	232.4 ± 13.7	156.1 ± 10.9	247.0 ± 17.6
20%	Cellulose	241.3 ± 11.9	247.4 ± 23.2	270.6 ± 9.5	157.9 ± 10.0	174.2 ± 13.7
	Potato starch	244.4 ± 14.0	177.7 ± 16.1	220.2 ± 11.4	163.0 ± 15.0	192.3 ± 14.4
	Pectin	243.5 ± 13.8	234.3 ± 20.8	219.6 ± 7.3	140.9 ± 12.9	211.8 ± 39.2
Main effects (*P* values)						
Protein type		0.029	0.748	0.118	0.319	0.063
Protein level		0.023	0.295	0.128	0.384	< 0.001
Carbohydrates		0.666	0.143	0.070	0.005	0.539
Protein type x Protein level		0.301	0.643	0.702	0.516	0.474
Protein type x Carbohydrates		0.427	0.176	0.018	0.135	0.443
Protein level x Carbohydrates		0.740	0.416	0.251	0.188	0.040
All		0.870	0.725	0.879	0.798	0.546

### DNA damage

The extent of DNA damage in colonic epithelial cells, expressed as ‘Tail Intensity Units’, was affected by protein level in the diet (Table 6). Rats fed HP diets had less DNA damage compared to rats fed LP diets (*P* < 0.001). Significant effects of interaction between protein level and carbohydrates was observed (*P* = 0.040). In rats fed LP diets, DNA damage was greater in rats fed cellulose than in those fed pectin, whereas in rats fed HP diets, the least DNA damage occurred in rats fed cellulose.

DNA damage in colonocytes was inversely correlated with the relative weight of cecal digesta (*r* = –0.64, *P* = 0.026), and directly proportional to cecal concentration (*r* = 0.62, *P* = 0.030) and cecal pool of phenol (*r* = 0.60, *P* = 0.038).

A summary of the effects of experimental factors on parameters related to large intestine health in rats is presented in Table 7.

**Table 7:** Summary of the effects of the experimental factors on biomarkers of the large intestine health in rats. The changes (↑ increase or ↓ decrease) in the parameters are shown in relation to casein diets, lower-protein diets, and cellulose, respectively. PPC–potato protein concentrate, CAS–casein, HP–higher protein diet, LP–lower protein diet, P—pectin, RPS—resistant potato starch.

Biomarker	Experimental factors		
	PPC vs CAS	HP vs LP	Carbohydrates
Cecal tissue	↑		↑ P
Cecal digesta	↑	↑	↑ P
Digesta pH			↓ P, ↓ RPS
Acetic acid:			
concentration		↓	
pool	↑		
Propionic acid:			
concentration		↓	
Isobutyric acid:			
pool	↑	↑	
Isovaleric acid:			
pool	↑	↑	
Total SCFA:			
concentration		↓	
Phenol:			
concentration	↓		
p-Cresol:			
Pool	↑		
Ammonia	↑		↑ P, ↑ RPS
Cecal crypts	↑	↓	
Colonic myenteron			↓ P
Colonocyte DNA damage		↓	

## Discussion

Potato protein is known to be more resistant to digestion than casein, and therefore decreases body weight gain in growing rats [12]. Older animals, used in our study, seemed to be insensitive to the lower digestibility of PPC, thus its negative impact on growth was not observed. However, feeding PPC and HP diets increased the relative weight of cecal digesta in rats, suggesting a greater influx of non-digested protein and its accumulation in the cecum. Indigestible carbohydrates also affected cecal parameters. Pectin seemed to exert the strongest effect on the relative weight of cecal digesta and tissue, which can be attributed to its greater fermentability in comparison to other carbohydrates [7]. Correlations between relative cecal weight, relative weight of cecal content, and digesta pH may suggest that a more acidic environment, caused by indigestible carbohydrate fermentation, contributed to cecal enlargement, which was also found in other studies [12, 13].

Our findings regarding the cecal pool of SCFAs confirm earlier studies related to the lower digestibility of potato protein [12, 14] and suggest that its fermentation leads to a greater production of acetic, isobutyric, and isovaleric acids. Acetic acid can originate from dietary fiber and non-essential amino acids, while isoacids are the end-products of bacterial catabolism of essential amino acids, i.e. valine and leucine [15]. The greater cecal pool of BCFA in rats fed HP diets can be attributed to a greater intake of these branched-chain amino acids.

The negative correlations between the relative weight of cecal digesta, concentrations of propionic and butyric acids, and the sum of SCFA probably result from the dilution effect of cecal digesta, since the highest concentration of SCFA was found in rats with the smallest amount of digesta. Our finding that indigestible carbohydrates and protein type had no effect on SCFA concentration was unexpected. This is in contrast to other experiments on rats, in which cecal fermentation of protein escaping digestion in the small intestine, resulted in enhanced production of BCFA [1]. In other studies, diets containing pectin contributed to a greater production of acetic acid [7], while resistant starch increased butyric acid production [8]. Pectin and starch differ in the fermentation pathway [16]. The former is catabolized in the pentose phosphate pathway and the latter in the glycolytic pathway. Many of the intestinal anaerobic bacteria utilize the flexibility of different fermentation routes, which enables them adaptation of substrate catabolism efficiency in response to changing environmental conditions [16]. Interactions between protein type, protein level, and indigestible carbohydrates reflect a high complexity of microbiological processes, in response to the composition of digesta entering the large intestine. As shown by the interaction, there is an interplay of dietary compounds (protein and carbohydrates) on SCFA concentration, which is the result of bacterial production and utilization by intestinal tissue [17]. This interplay may be a consequence of changes in the composition of microflora due to dietary factors. CAS diets have been shown to exert a proliferative effect on *Bifidobacterium* [18], which effectively degrade resistant starch [19]. However, it is thought that bacteria of the *Firmicutes* and *Bacteroidetes* phyla play a major role in polysaccharide degradation. *Firmicutes* include *Butyrivibrio* and *Ruminococcus* spp., the growth of which is stimulated by high levels of dietary fiber [20]. Other cellulolytic bacteria of this phylum belong to the *Clostridium* and *Eubacterium* spp. [19], and it is possible that they also contributed to the SCFA profile observed in rats fed cellulose in the present study. *Bacteroides* sp. also exhibit cellulolytic activity, however these members of the *Bacteroidetes* phylum are thought to play the most important role in pectin degradation [20]. In addition, the introduction of complex carbohydrates of plant origin to the diet increases the abundance of *Bacteroidetes* [21]. Other bacteria that can utilize pectin as an energy source belong to *Bifidobacterium* sp., *Lactobacillus* sp., *Enterococcus* sp., *Clostridium* sp., and *Enterobacteriaceae* [20]. Although the composition of cecal microflora was not investigated in our study, we can assume that different dietary treatments affected various bacterial populations and thus contributed to the observed fermentation patterns. This assumption is consistent with recent evidence indicating positive correlations between the main SCFA (acetic, propionic, and butyric acids), *Bacteroidetes*, and *Verucomicrobia* levels, and negative correlation between SCFA and *Firmicutes* levels [21]. The disparity of some of our observations with other studies on rats can be attributed to differences between strains of rats and thus the microbiological status of the animals, diet compositions, age, and duration of the experiments [22].

Protein fermentation is detrimental to the host’s health, as it is involved in the development of colon cancer, ulcerative colitis, and aging. During putrefaction, a wide range of potentially toxic metabolites is formed, such as phenol and p-cresol, from bacterial degradation of aromatic amino acids [23]. Cresols are produced from tyrosine by *Bacteroides fragilis* and different species of *Clostridium* and *Bifidobacterium*, whereas phenol production is attributed to e.g. *E*. *coli* [24]. Contrary to the expectations, in our study, rats fed PPC had a smaller concentration of phenol in cecal digesta compared to those fed CAS. Potato protein digestibility is lower than that of casein, thus the flow of undigested protein from the ileum to the cecum, and its availability for bacterial fermentation is greater. Therefore, proteolysis should be intensified and it is difficult to explain why this effect of PPC was observed only in case of the cecal pool of p-cresol. A shift in the cecal microflora from phenol-producing to cresol-producing bacteria in rats fed PPC diets may provide a possible explanation. The negative correlation between phenol concentration and the relative weight of cecal digesta may suggest that greater digestibility of casein and thus, the smaller relative weight of cecal digesta, favors phenol production.

β-glucuronidase activity is a biomarker for the risk of carcinogenesis, based on the hydrolysis of glucuronide conjugates and the activation of carcinogens and toxins in the large intestine [25]. Dietary fiber may reduce the risk of the development of colon cancer by its inhibitory effect on this enzyme’s activity [26]. The beneficial effects of RPS on β-glucuronidase activity observed in our study, is consistent with previously reported results [1]. The mechanism behind these effects remains unclear, but may be related to changes in microflora composition induced by various dietary treatments. Some bacterial species including *Bacteroides* [27], *Bifidobacterium*, *Eubacterium*, *Ruminococcus*, *E*. *coli*, *Lactobacillus*, *Staphylococcus*, and *Clostridium* [25], exhibit high levels of β-glucuronidase activity, and any shifts in their populations due to experimental factors must be considered.

Ammonia originates predominantly from bacterial deamination of amino acids and to a lesser extent from hydrolysis of blood urea. Ammonia concentration in digesta results from its production and utilization by bacteria in protein synthesis, and its absorption by intestinal epithelium [23]. The effects of a higher protein intake in humans [28, 29] and rats [30] observed in previous reports, were not observed in the present study. The large disparities in experimental conditions make the results difficult to compare, and the mechanisms by which protein levels affect ammonia concentration remain unclear. Our study revealed, however, that colonic ammonia concentrations depend on protein type in the diet, and is greater in rats fed PPC than in those fed CAS. Potato protein is characterized by a lower digestibility compared to that of CAS, and may intensify proteolytic fermentation, reflected by the increase in the cecal pool of p-cresol. Although BCFA and phenolic compounds, which are biomarkers of proteolysis, were not analyzed in colonic digesta, our results suggest that protein, which has escaped digestion in the small intestine, is degraded by microbiota of the colon, leading to the production of ammonia. Our results also demonstrate that colonic ammonia concentration correlates negatively with cecal digesta pH; correlates positively with the relative weight of cecal tissue; and is reduced by cellulose in comparison to pectin and RPS. A previous study reported that cellulose decreases ammonia concentration in the distal colon of rats and reduces cecal surface area, when compared to pectin [31]. Previously reported results, along with our findings, indicate that the inhibitory effect of cellulose on ammonia concentration in the colon probably starts in the cecum. However, due to technical limitations, cecal ammonia levels could not have been analyzed in the present study and this hypothesis requires further study for confirmation. Nevertheless, feeding cellulose decreases the amount of cecal digesta and cecal tissue weight, and because of its low fermentability, increases cecal digesta pH. The reduced cecal tissue weight corresponds well with a smaller absorptive area, which contributes to a greater influx of unabsorbed ammonia into the colon. Cellulose may also increase the pH of colonic digesta, thus facilitating the pH-dependent absorption of ammonia [31]. Increased absorption by epithelial cells leads to a lower ammonia concentration in the digesta of the large intestine and a greater blood urea concentration [32].

Morphology of the intestine is a component of gut health that is related to the absorption and metabolism of nutrients. Abnormal changes of morphological parameters in response to dietary treatments may increase susceptibility to functional disorders [33]. In the present study, protein type and level affected cecal crypt depth, whereas colonic crypt depth was affected by the interaction between protein type and carbohydrates. Rats fed PPC and LP diets had deeper cecal crypts in comparison to those fed CAS and HP diets. This can be attributed to more intensive cell proliferation and renewal, processes that are necessary to replace old epithelial cells [33]. On the other hand, hyperproliferation (considered a marker for the risk of cancer development), is initiated by early death of surface colonocytes, damaged through contact with cytolytic components of colonic digesta [34]. The exact mechanism underlying the effects of PPC and LP diets on cecal crypts remains unclear, but can be attributed to the production of fermentation end-products. The positive correlation between colonic crypt depth and the concentration of butyric acid in the cecum may suggest a trophic effect of butyric acid. This effect can be attributed to the activity of glucagon-like peptide-2 (GLP-2), which modulates growth and functions of the intestine and regulates carbohydrate metabolism [35]. SCFAs increase GLP-2 secretion in the gastrointestinal tract; however, its level in cecal and colonic tissue was not analyzed in the present study. Therefore, we can only speculate that cecal butyric acid may stimulate GLP-2 secretion in the colon.

The effect of experimental factors on myenteron thickness was found only in the colon, where it was increased by feeding cellulose, compared to pectin. Greater myenteron thickness is reported to correspond with increased intestinal motility [36]. Therefore, myenteric hypertrophy in the colon can also enhance the effects of cellulose on ammonia concentrations, by facilitating the removal of ammonia from the large intestine through enhanced intestinal motility. This assumption is supported by significant correlations between myenteron thickness in the colon, cecal parameters, and ammonia concentration.

In contrast to previous studies [4, 8], rats fed HP diets had less DNA damage compared to rats fed LP diets. Potato starch had no protective effect on colonocyte DNA, which is consistent with the findings of previous reports [27]. Resistant starch exhibited beneficial effects to rats fed at a higher protein level than that used in our study [37], and which exceeded nutritional requirements. These findings suggest that resistant starch is beneficial to colonic health when dietary protein level is higher than 20%. The lack of RPS effects may also be explained by its difference in chemical properties and lower dietary content, compared to high amylose corn starch investigated in other studies [4, 37]. Feeding rats with diets that lower the cecal digesta weight contributes to greater phenol concentrations and thus, greater DNA damage of colonocytes. Genotoxic effects of phenolic compounds can be associated with the production of N-nitroso compounds and mutagenic diazoquinone [5], and with the reduction of cell viability and increase in membrane permeability [23]. In addition, presumable reduction of bacterial cell numbers may contribute to increased DNA damage in colonocytes by a mechanism associated with local vitamin B_12_ (cobalamin) deficiency. Biosynthesis of vitamin B_12_ is restricted to bacteria [38]; therefore, nutritional factors reducing their population in the large intestine may contribute to lower levels and bioavailability of this vitamin. Cobalamin deficiency mimics the effect of radiation on DNA, causing oxidative damage, and single and double strand breaks. Effects of the deficiency are similar to those of folate deficiency. Chromosome breakage occurs because of impaired methylation of uracil to thymine, followed by incorporation and accumulation of uracil in DNA. Uracil must be subsequently excised by a repair enzyme, which leads to DNA strand breaks [39]. Although vitamin B_12_ was provided in a vitamin premix in all experimental diets in the present study, its supply could have been insufficient in light of possible reductions in its bacterial biosynthesis in rats fed LP diets.

## Conclusions

The results of the present study were not always consistent with previous reports, and the effects of experimental factors on parameters related to gut health are inconclusive. However, cecal parameters showed more intense fermentation of pectin and potato protein, than cellulose and casein, respectively. Higher dietary protein levels also exhibited a stimulatory effect on cecal parameters. Concentrations of SCFA were affected by interactions among experimental variables, suggestive of the modifying effects of nutritional factors on the fermentation process and possibly, on bacterial populations in the gut. Different types of protein affected the concentration of metabolites in large intestinal compartments differently. Feeding potato protein reduced phenol concentrations in the cecal digesta, but increased both the cecal pool of p-cresol, and the concentration of ammonia in the colon. Our observations on colonocyte DNA damage may suggest the beneficial influence of a protein level of 20% in the diet of rats. Moreover, experimental factors influenced large intestine health by its effects on cecal digesta weight, which directly or indirectly determines the production of fermentation end-products, morphological parameters, and DNA status in colonocytes. Nutritional factors increasing the cecal digesta weight contribute to smaller concentrations of phenol (potato protein) and less DNA damage (higher protein level), but may be undesirable because of increased levels of ammonia in the colon (potato protein and pectin). Owing to the differences in the action of experimental factors on particular indices, our results obtained using the rat model require further evaluation, and remain to be confirmed in human studies before the translations in nutritional recommendations to improve the health status of the large intestine.

## Supporting Information

S1 Dataset Raw Data. (XLSX)
